# Supplementary material for: The hammam effect or how a warm ocean enhances large scale atmospheric predictability
Source: Nat Commun. 2019 Mar 21;10:1316. doi: 10.1038/s41467-019-09305-8 (PMC6428824; doi:10.1038/s41467-019-09305-8)
Supplement: Supplementary file 1 — Supplementary Material [file 41467_2019_9305_MOESM1_ESM.pdf]

Supplemental material: The hammam effect or how a warm ocean enhances large-scale atmospheric predictability

Davide Faranda[1,2], M. Carmen Alvarez-Castro[1,3], Gabriele Messori[1,4,5], David Rodrigues[1] & Pascal Yiou[1]

[1] *Laboratoire des Sciences du Climat et de l'Environnement LSCE-IPSL, CEA Saclay l'Orme des Merisiers, UMR 8212 CEA-CNRS-UVSQ, Université Paris-Saclay, 91191 Gif-sur-Yvette, France*

[2] *London Mathematical Laboratory, 8 Margravine Gardens, London, United Kingdom*

[3] *Climate Simulation and Prediction Division, Centro Euro-Mediterraneo sui Cambiamenti Climatici, Bologna, Italy.*

[4] *Department of Earth Sciences, Uppsala University, Uppsala, Sweden*

[5] *Department of Meteorology, Stockholm University and Bolin Centre for Climate Research, Stockholm, Sweden*

Supplementary Material:

- Supplementary Note 1
- Supplementary Figures
- Supplementary Tables
- Supplementary References

Supplementary Note 1

The Mann Kendall Trend Test is a non-parametric test, i.e. it does not assume a specific underlying probability distribution. The null hypothesis (H_0) for this test is that there is no monotonic trend in the time series. The alternate hypothesis H_1 is that a trend exists. This trend can be positive, negative, or non-null. For a time series X_i, $i = 1, \ldots, n$, the Mann-Kendall statistic is computed as:

$$S = \sum_{k=1}^{n-1} \sum_{j=k+1}^{n} \mathrm{sgn}(X_j - X_k)$$

where sgn is the sign function. If $n < 10$, the value of $|S|$ is compared directly to the theoretical distribution of S derived by Mann and Kendall [1]. Here, the two-tailed test is used. At a given probability level, H_0 is rejected in favor of H_1 if the absolute value of S equals or exceeds a specified value $S_{\alpha/2}$. A positive (negative) value of S indicates an upward (downward) trend. The Mann-Kendall test does not check for the sign of the trend. For $n \geq 10$, the statistic S is approximately normally distributed with zero mean and known variance. Its values can be used to compute a test-statistics [2].

Supplementary Figures

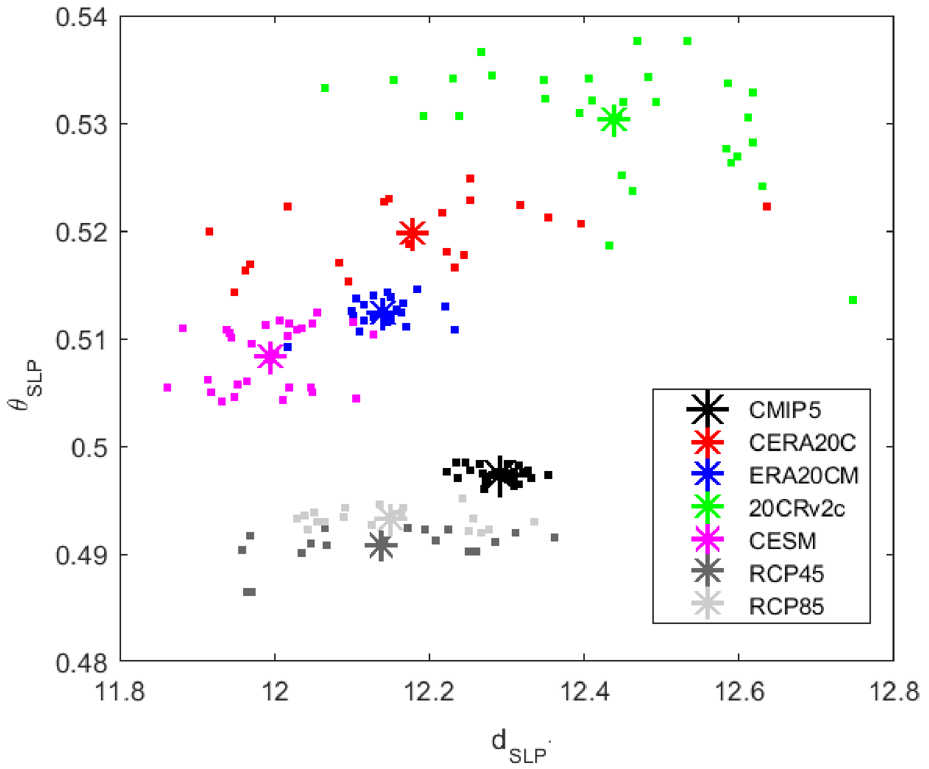

Supplementary Figure 1: Absolute mean values of local dimension and inverse persistence for all datasets. Absolute mean values of local dimension d_{SLP} and inverse persistence θ_{SLP} for single members or models (squares) and means of the ensembles (stars). Different colors correspond to different datasets as shown in the legend.

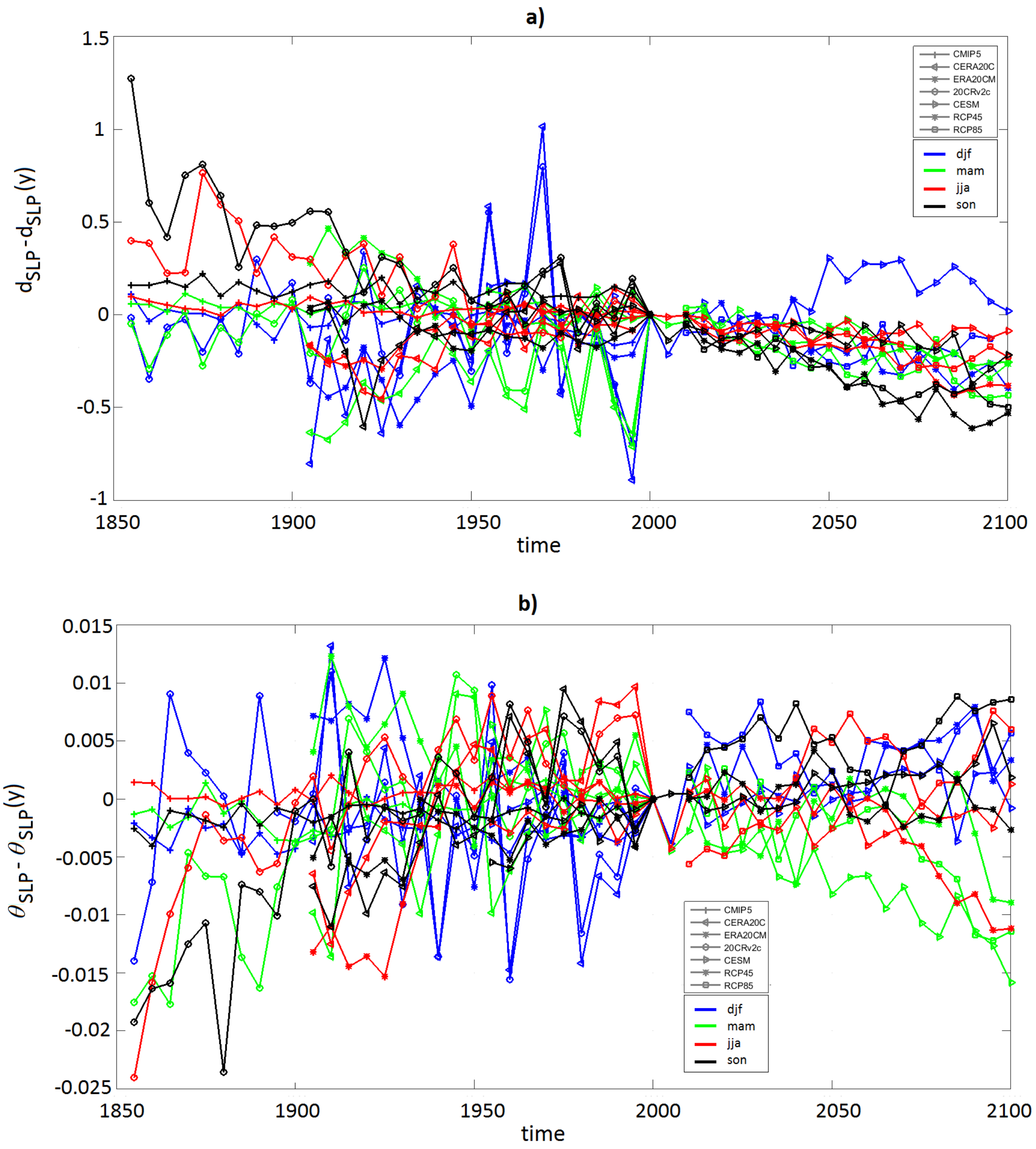

Supplementary Figure 2: Local dimension and inverse persistence for all the SLP datasets, divided by season. 5-year averages of local dimension d_{SLP} (a) and inverse persistence θ_{SLP} (b) minus the values $d_{\mathrm{SLP}}(y)$ and $\theta_{\mathrm{SLP}}(y)$ with $y = 2000$ (or $y = 2006$ for the RCP scenarios). Different colors correspond to different seasons as shown in the legend. Symbols correspond to different datasets as shown in the legend.

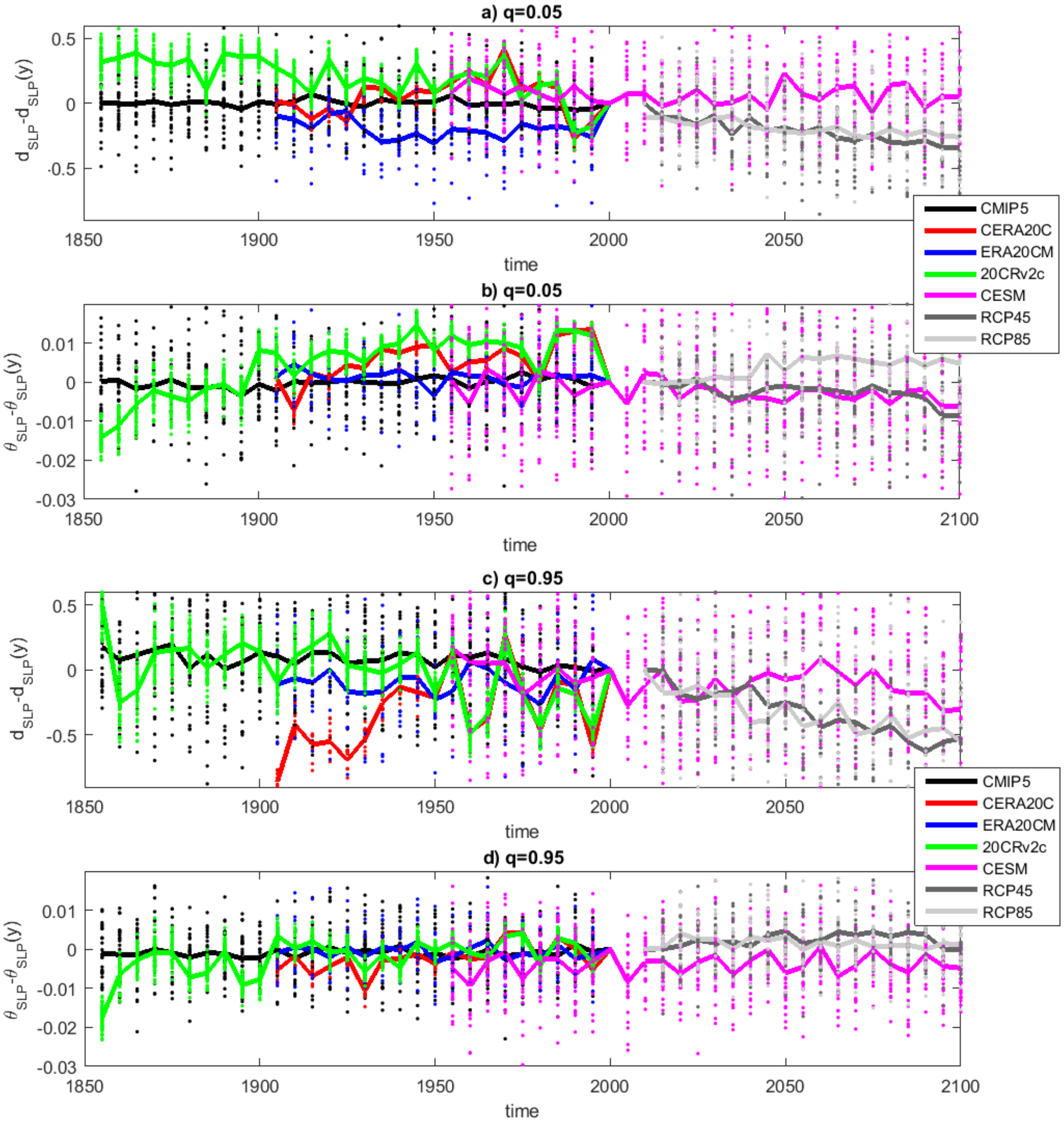

Supplementary Figure 3: 5th (a,b) and 95th (c,d) percentiles of local dimension and inverse persistence for all the datasets. 5-year averages of local dimension d_{SLP} (a,c) and inverse persistence θ_{SLP} (b,d) minus the values $d_{\mathrm{SLP}}(y)$ and $\theta_{\mathrm{SLP}}(y)$ with $y = 2000$ (or $y = 2006$ for the RCP scenarios). Different colors correspond to different datasets as shown in the legend. Dots: single member or model. Solid lines: means of the ensembles.

Supplementary Tables

Ensemble	Type	Period	Ens. Size	Simulations<br>Hist.	 RCP45	 RCP85	 AMIP/4K/4CO2
20CRv2c	Reanalysis	1850-2000	56	X			
ERA20CM	Reanalysis	1900-2000	10	X			
CERA20C	Reanalysis	1900-2000	10	X			
CESM	Model	1950-2100	32	X	X	X	
CMIP5	Model		CMCC-CESM	X			
		hist:1850-2000	CanESM2	X			
		rcp45:2006-2100	CMCC-CMS	X			
		rcp85:2006-2100	MIROC-ESM-CHEM	X	X	X	
		amip:1979-2005	MIROC-ESM	X	X	X	
			BCC-CSM1	X	X	X	X
			IPSL-CM5B	X			X
			NorESM1-M	X	X	X	
			FGOALS-G2	X	X	X	X
			MPI-ESM-P	X			
			MPI-ESM-LR	X			X
			CSIRO-MK3-6-0	X			
			CMCC-CMS	X	X	X	
			MPI-ESM-MR	X	X	X	X
			IPSL-CM5A	X	X	X	X
			INMCM4	X			
			ACCESS 1-0	X			
			MIROC5	X			X
			CNRM-CM5	X	X	X	X
			MRI-ESM1	X			
			BCC-CSM1-M	X			
			MRI-CGCM3	X	X	X	X
			EC-EARTH	X			
			CESM1-FASTCHEM	X			
			CESM1-CAM5	X			
			CESM1-BGC	X			
			CCSM4	X			
			BNU-ESM		X	X	
			GFDL CMR3		X	X	
			GFDL ESM2G		X	X	
			GFDL ESM2M		X	X	
			HadGEM2 CC		X	X	
			HadGEM2 ES		X	X	
			HadGEM2-A		X	X	X
			IPSL CM5A MR		X		

Supplementary Table 1: List of datasets analysed. Crosses indicate the types of runs discussed in the text.

Ensemble	Winter (DJF) H	$pvalue$	trend	Spring (MAM) H	$pvalue$	trend	Summer (JJA) H	$pvalue$	trend	Fall (SON) H	$pvalue$	trend
20CRv2c	0	0.94	$=$	0	0.54	$=$	1	6.9e-6	$\Downarrow$	1	4.0e-7	$\Downarrow$
ERA20CM	1	0.007	$\Uparrow$	1	0.0086	$\Downarrow$	1	0.006	$\Uparrow$	1	0.03	$\Downarrow$
CERA20C	0	0.38	$=$	0	0.13	$=$	1	3.1e-4	$\Uparrow$	1	0.048	$\Uparrow$
CESM	0	0.06	$=$	1	4.86e-9	$\Downarrow$	1	4.9e-6	$\Downarrow$	1	1.4e-6	$\Downarrow$
CMIP5	1	0.008	$\Downarrow$	1	0.05	$\Downarrow$	1	0.004	$\Downarrow$	1	0.03	$\Downarrow$
RCP45	0	0.73	$=$	1	3.6e-05	$\Downarrow$	1	4.6e-4	$\Downarrow$	1	5.4e-6	$\Downarrow$
RCP85	1	1.2e-4	$\Downarrow$	1	2.7e-4	$\Downarrow$	1	2.8e-6	$\Downarrow$	1	1.9e-6	$\Downarrow$

Supplementary Table 2: Mann-Kendall test p-values for d_{SLP} in each season. Arrows $\Downarrow$/$\Uparrow$ denote significant decreasing/increasing trends at the 5% level, respectively. The equal sign $=$ denotes absence of significant trends.

Ensemble	Winter (DJF) H	$pvalue$	trend	Spring (MAM) H	$pvalue$	trend	Summer (JJA) H	$pvalue$	trend	Fall (SON) H	$pvalue$	trend
20CRv2c	0	0.32	$=$	1	1.5e-4	$\Downarrow$	1	4.9e-06	$\Uparrow$	1	2.4e-06	$\Uparrow$
ERA20CM	1	0.001	$\Downarrow$	1	0.009	$\Downarrow$	1	0.01	$\Uparrow$	0	0.1	$=$
CERA	0	0.67	$=$	0	0.18	$=$	1	8.6e-05	$\Uparrow$	1	8.3e-4	$\Uparrow$
CESM	1	0.007	$\Uparrow$	1	2.0e-09	$\Uparrow$	0	0.69	$=$	1	1.4e-08	$\Uparrow$
CMIP5	0	0.32	$=$	1	0.004	$\Uparrow$	0	0.39	$=$	0	0.12	$=$
RCP45	0	0.73	$=$	1	3.6e-4	$\Downarrow$	1	0.002	$\Uparrow$	1	0.036	$\Uparrow$
RCP85	1	0.04	$\Uparrow$	0	1	$=$	1	8.9e-05	$\Downarrow$	1	0.05	$\Downarrow$

Supplementary Table 3: Mann-Kendall test p-values for θ in each season. Arrows $\Downarrow$/$\Uparrow$ denote decreasing/increasing trends at the 5% level, respectively. The equal sign $=$ denotes absence of significant trends.

Supplementary References

[1] Yue, S., Pilon, P., Cavadias, G. (2002). Power of the MannKendall and Spearman's rho tests for detecting monotonic trends in hydrological series. Journal of hydrology, 259(1-4), 254-271.

[2] Motiee H., McBean E., 2009. An Assessment of Long Term Trends in Hydrologic Components and Implications for Water Levels in Lake Superior. Hydrology Research, 40.6, 564-579.
